# Association between underweight status or low body mass index and the risk of developing superior mesenteric artery syndrome following scoliosis corrective surgery in pediatric patients: a review of the literature

**DOI:** 10.1007/s43390-024-00929-5

**Published:** 2024-07-24

**Authors:** Lyssa Lamport, Jon-Paul DiMauro, Stephani Johnson, Susan Roberts, Jane Ziegler

**Affiliations:** 1 Cohen Children’s Medical Center of New York, New Hyde Park, New York, USA; 2 Zucker School of Medicine at Hofstra/Northwell, Orthopedic Surgery, Hempstead, New York USA; 3 Department of Clinical and Preventive Nutrition Sciences, School of Health Professions, Rutgers, The State University of New Jersey, Newark, New Jersey USA

**Keywords:** Superior mesenteric artery syndrome, Wilkie’s syndrome, Duodenal compression, Scoliosis, Idiopathic scoliosis, Spinal fusion, Body mass index, Underweight, Malnutrition

## Abstract

Superior mesenteric artery (SMA) syndrome is the compression of the third portion of the duodenum between the abdominal aorta and the superior mesenteric artery. Although multifactorial, the most frequent cause of SMA syndrome is significant weight loss and cachexia often induced by catabolic stress. SMA syndrome resulting from scoliosis surgery is caused by a reduction of the aortomesenteric angle and distance. Risk factors include rapid weight loss, malnutrition, and a rapid reduction in the mesenteric fat pad and are the most common causes of a decrease in the aortomesenteric angle and distance. Surgically lengthening the vertebral column can also lead to a reduction of the aortomesenteric distance, therefore, has been identified as a risk factor unique to spinal surgery. Despite a reported decline in SMA syndrome cases due to improved surgical techniques, duodenal compression is still a risk and remains a life-threatening complication of scoliosis surgery. This article is a cumulative review of the evidence of being underweight or having a low body mass index as risk factors for developing SMA syndrome following surgical scoliosis instrumentation and correction.

## Introduction

Superior mesenteric artery (SMA) syndrome is a medical condition characterized by the compression of the third portion of the duodenum between the abdominal aorta and the superior mesenteric artery [1, 2]. SMA syndrome is alternatively known by several names including chronic duodenal ileus, Wilkie syndrome, arterio-mesenteric duodenal compression syndrome, and cast syndrome [1, 2]. The earliest documented case of SMA syndrome was reported by Carl Von Rokitansky in 1842 [1–3]. Subsequently, in 1927, further research by Carl Wilkie contributed to the understanding of the pathophysiology and diagnostic features of SMA syndrome [1–3].

**Fig. 1:**
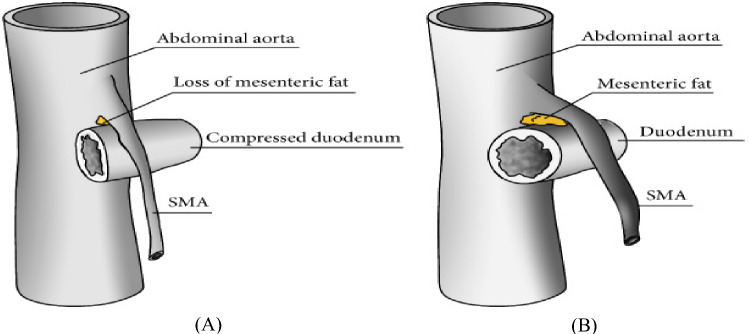
A schematic view of the aorta, SMA, and third portion of the duodenum. **A** Loss of mesenteric fat producing an acute angle between the aorta and SMA and compression of the duodenum. **B** Presence of mesenteric fat and a normal angle between aorta and SMA

SMA syndrome occurs when there is a reduction in the mesenteric fat pad located between the aorta and superior mesenteric artery [2–5]. This reduction leads to a narrowing of the angle between these two vessels, called the aortomesenteric angle [2, 4, 5]. The mesenteric fat pad plays a critical role as a cushion, supporting the SMA and preventing it from pressing against the spine, thereby averting compression of the duodenum [3–6]. The aortomesenteric angle is the angle between the aorta and the SMA and falls within the range of 38–65 degrees [4–6]. However, when the aortomesenteric angle decreases to less than 25 degrees, compression of the third part of the duodenum occurs (Fig. 1) [4–6]. The likelihood of developing SMA syndrome has been linked to significant weight loss and cachexia, frequently resulting from catabolic stress [3, 4, 6, 7]. Additional risk factors found in the medical literature encompass surgical correction of scoliosis, hypertrophic ligament of Treitz, peritoneal adhesions, duodenal malrotation, Ladd’s bands, abdominal aortic aneurysm, lumbar hyperlordosis, and mesenteric root neoplasm [3, 7, 8].

Diagnosing SMA syndrome can be challenging because symptoms tend to be non-specific and can develop acutely or gradually [1, 2, 7, 9, 10]. The most frequent presentation is epigastric pain, nausea, and vomiting [2, 3]. Other reported symptoms include abdominal distention, weight loss, early satiety, and postprandial epigastric pain that worsens when the patient is in a supine position [7, 9, 10]. The pain can become intermittent or chronic based on the severity of obstruction or compression [3, 6, 7, 10]. Chronic cases tend to cause intermittent symptoms that come and go. [7, 9, 10] This intermittent cycle of symptoms often leads to inadequate intake and weight loss which further aggravates the syndrome [7, 9, 10]. In more severe, acute cases, the obstruction and compression result in the dilation of the stomach which can be life-threatening if not treated [7, 9, 10].

Various imaging methods can be used to confirm the diagnosis of SMA syndrome [3, 11]. The most frequently used methods are X-ray and computed tomography (CT) scans [3, 11]. X-rays will show a dilated stomach and a diminished distal bowel gas [3, 11]. CT scan remains the gold standard as it allows for the visualization and measurement of the aortomesenteric angle, which is how SMA syndrome diagnosis is confirmed [3, 11]. Although limited research and not as commonly used, ultrasound imaging has been used to diagnose SMA syndrome [12, 13].

Due to the rarity of the syndrome, diagnosis is often made by symptoms, without radiographic confirmation; therefore, an accurate prevalence of the disease remains unknown. [3, 6, 9, 14] However, based on published case reports of confirmed SMA syndrome by radiographic testing, the incidence in the general surgical population is 0.13 to 0.78%, regardless of surgery type. [2, 10] SMA syndrome tends to occur in adolescents and young adults, ranging in age from ten to thirty-nine years old but can occur at any age. [14] It tends to occur more frequently in females than males at a ratio of 3:2. [14] No ethnic predisposition has been reported in the literature [14].

**Fig. 2:**
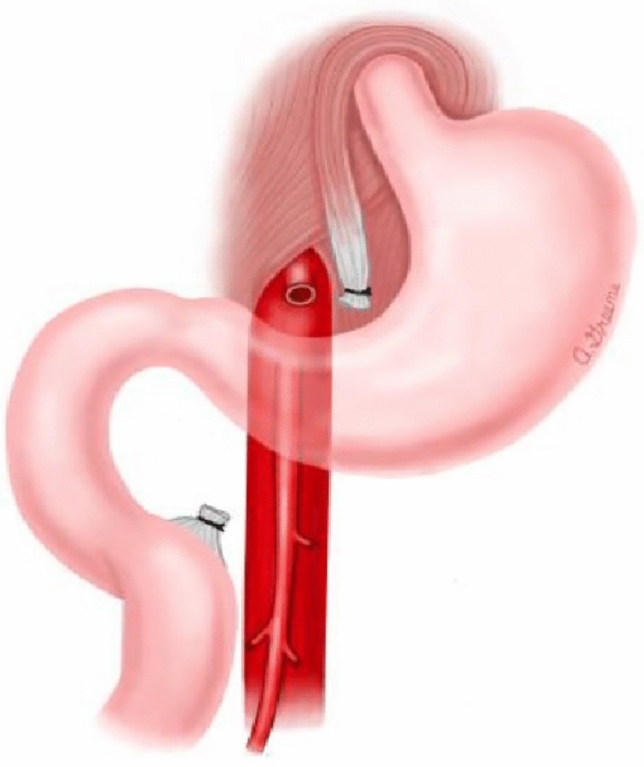
Strong’s procedure

**Fig. 3:**
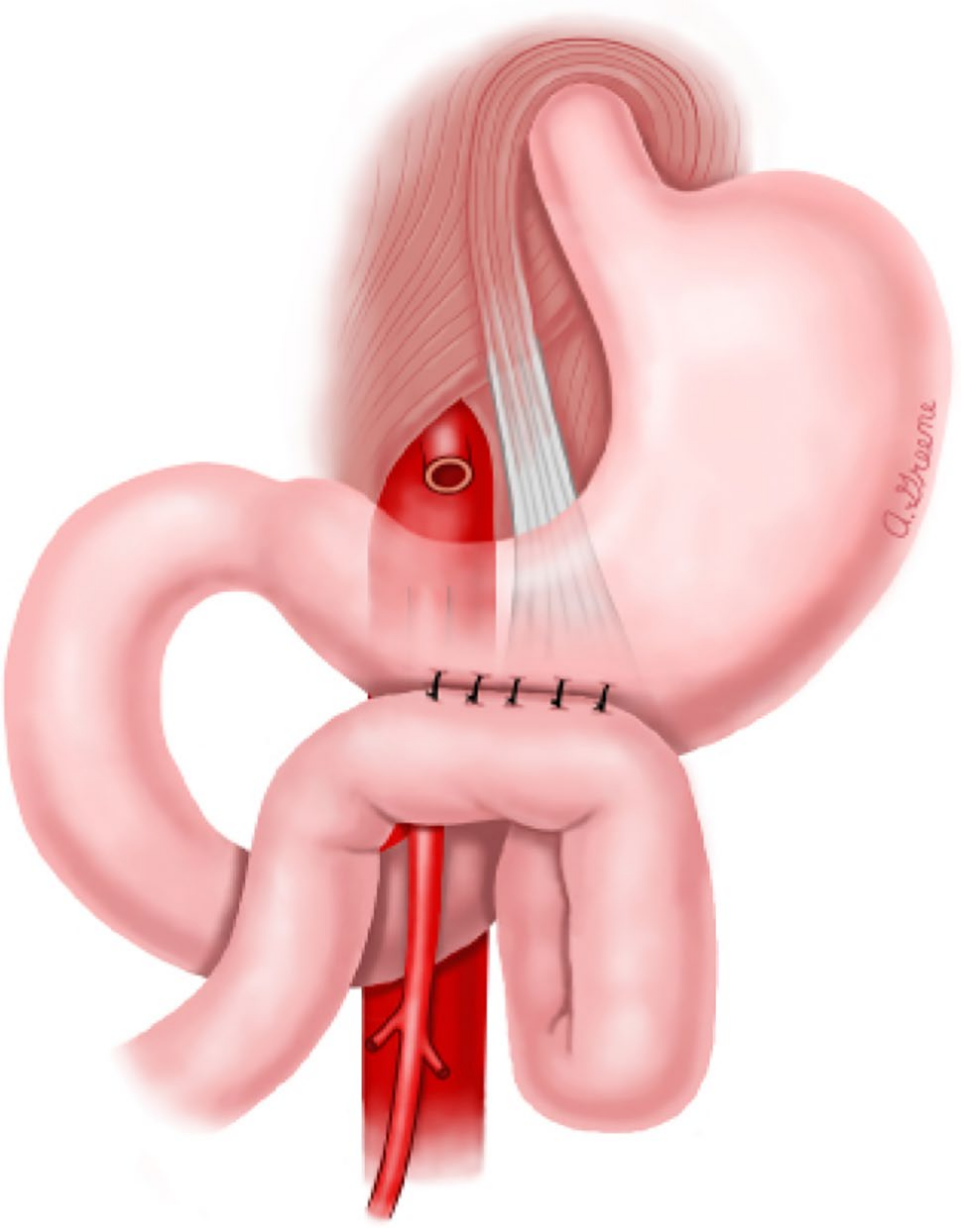
Gastrojejunostomy

**Fig. 4:**
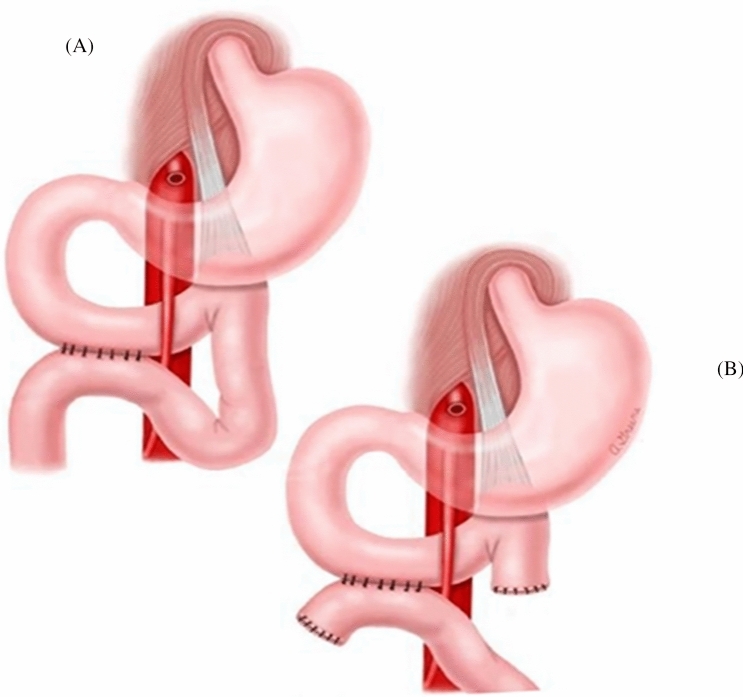
Latero-lateral duodenojejunostomy without separation of the 4th duodenal portion (**A**) and with separation (**B**)

To date, no published literature is available on nutrition strategies for the prevention of SMA syndrome. However, there is limited research on treatment strategies for SMA syndrome [3]. Management of SMA syndrome varies depending on the severity of the duodenal compression and the patient's ability to tolerate an oral diet [3, 15]. The main goal of treatment is to increase the mesenteric fat pad, to increase the aortomesenteric angle, thus improving symptoms [3, 15]. In the acute phase, initial nutrition management often includes fluid resuscitation, electrolyte correction, parenteral nutrition, and gastric decompression. [3, 15] If the patient can eat, small frequent, nutrient-dense meals or supplements are encouraged. [3, 15] In some instances, lying on the left lateral decubitus position has been shown to improve symptoms. [3, 15] However, positional management of SMA syndrome may not be conducive postoperatively for some surgical procedures, including scoliosis surgery. If conservative management fails, then surgical intervention is necessary. [3, 16, 17] Surgical procedures to repair SMA syndrome are Strong’s procedure, open or laparoscopic gastrojejunostomy, or duodenojejunostomy. [16–20] Strong’s procedure (Fig. 2) involves mobilization of the duodenum by division of the ligament of Treitz. [16–20] Strong’s procedure does not require anastomosis and is associated with shorter postoperative recovery; however, the procedure is not possible if the patient has adhesions or short vascular connections between the inferior pancreaticoduodenal artery and duodenum [16–18]. A gastrojejunostomy (Fig. 3) does not relieve duodenal obstruction but provides symptomatic relief of a distended stomach [17–20]. This type of procedure is considered if other surgical options prove too difficult. [16–20] Duodenojejunostomy (Fig. 4) is technically the most challenging procedure but is more physiologic than a gastrojejunostomy [16–18, 20]. This procedure involves releasing the compressed portion of the duodenum and creating an anastomosis between the duodenum and jejunum. [16–18] The duodenojejunostomy provides a functional bypass allowing for earlier return of bowel function and use [16–18]. The most common surgical treatment for SMA syndrome is laparoscopic duodenojejunostomy, as it has the best success rate as well as the most rapid recovery [3, 16–18, 20].

Surgical correction of scoliosis has been identified as a risk factor for the development of SMA syndrome as previously stated. [2, 5, 10, 14, 21–25] SMA syndrome that occurs as a consequence of scoliosis surgery is believed to stem from a decrease in the aortomesenteric angle and the aortomesenteric distance following the elongation of the vertebral column. [10, 14, 21, 23, 26, 27] Risk factors such as preoperative underweight status and/or low body mass index (BMI), postoperative weight loss, malnutrition, and the rapid reduction in the mesenteric fat pad have been identified as the most frequent causes of a decrease in the aortomesenteric angle and distance. [21, 27–29] Although data are limited, other reported risk factors are those with a stiffer thoracic curve (< 60% correction on bending radiographs), those with a laterally displaced lumbar curve (Lenke B or C), and kyphosis [25, 30].

In the pediatric and adult population, the incidence of SMA syndrome following surgical correction and instrumentation of scoliosis surgery ranges from 1% to 4.7% [27–31]. Most cases of SMA syndrome following scoliosis surgery, occur within the first week postoperatively [21, 27–31].

Although the etiology of scoliosis is unclear, males and females tend to be thinner and have significantly lower body weight, BMI, and BMI-for-age/sex percentiles [32–34]. Approximately 21–26% of females with idiopathic scoliosis are reported to have a BMI < 17.5 kg/m^2^ or < 50th percentile gender-specific BMI-for-age. [32–34] Past studies have shown that individuals with idiopathic scoliosis tend to be more slender than their age-matched controls and have less fat mass [32, 33].

Following any surgical procedure, individuals often experience rapid or unintentional weight loss due to various factors, including catabolic stress, the recovery process, reduced hunger, and postoperative complications. [34, 35] Rapid postoperative weight loss can lead to a reduction in the mesenteric fat pad, thus exacerbating the risk of SMA syndrome. [34, 35] A study by Tarrant et al. showed that a presurgical low BMI compounded by greater than 10% postoperative weight loss following scoliosis surgery resulted in an increase in postoperative complications, including SMA syndrome. [34, 35] Tarrant et al. also concluded that a low BMI was independently associated with developing SMA syndrome. [35] Okur et al. conducted a study [*N* = 524] evaluating the relationship between BMI and the aortomesenteric angle where the duodenum crosses the aorta and SMA. [36] They found a positive correlation between BMI and the aortomesenteric angle (*P* < 0.001). [36] Ozkurt et al. showed that as the BMI decreases, the aortomesenteric angle does as well. [36] Therefore, the predisposition toward being underweight in conjunction with postoperative weight loss from catabolic stress and the typical recovery process puts scoliosis surgical patients at a greater risk for developing SMA syndrome [22–24, 26, 34, 35].

Considerable attention has also been directed toward SMA syndrome in individuals undergoing surgical spinal correction of scoliosis and the consequence of a rapid increase in trunk height. [22, 23] Individuals with scoliosis often exhibit a lower body weight relative to their height [22]. This height-to-weight disparity is exacerbated when these individuals undergo corrective procedures with instrumentation that abruptly increases their trunk length and overall height. [22] Surgical correction of scoliosis significantly elongates the spinal column and increases external compression of the distal duodenum as the distal duodenum goes through a tapered angle which is formed of the aorta, the anterior wall of the spinal column, and the posterior wall of the superior mesenteric artery [23, 37]. Due to scoliosis correction surgery, lateral mobility of the SMA is decreased and so the aorto-mesenteric angle is changed. It is observed that the elongation of the spinal column, especially in the lumbar region, after the surgery, is an important risk factor for developing SMA syndrome. [22, 23, 37] Of note, SMA syndrome is a known complication of kyphosis correction surgery as well. [25, 38, 39] Surgical correction of kyphosis also involves lengthening the spine, resulting in a rapid increase in trunk and overall height, similar to scoliosis correction. [25, 38, 39] Scoliosis and kyphosis are often seen together; therefore, for this review, a diagnosis of kyphosis was included.

Since the 1990s, advancements in surgical techniques in spinal corrective procedures of scoliosis patients have led to decreased complication rates, including reduced incidence of SMA syndrome. [25] However, it is essential to recognize that despite a decline in confirmed SMA syndrome cases, due to these improved techniques, duodenal compression is still a risk and remains a life-threatening complication of scoliosis surgery [25].

This literature review aims to examine the evidence of being underweight or having a low BMI as a risk factor for developing SMA syndrome following surgical scoliosis correction.

### Search strategy

**Fig. 5:**
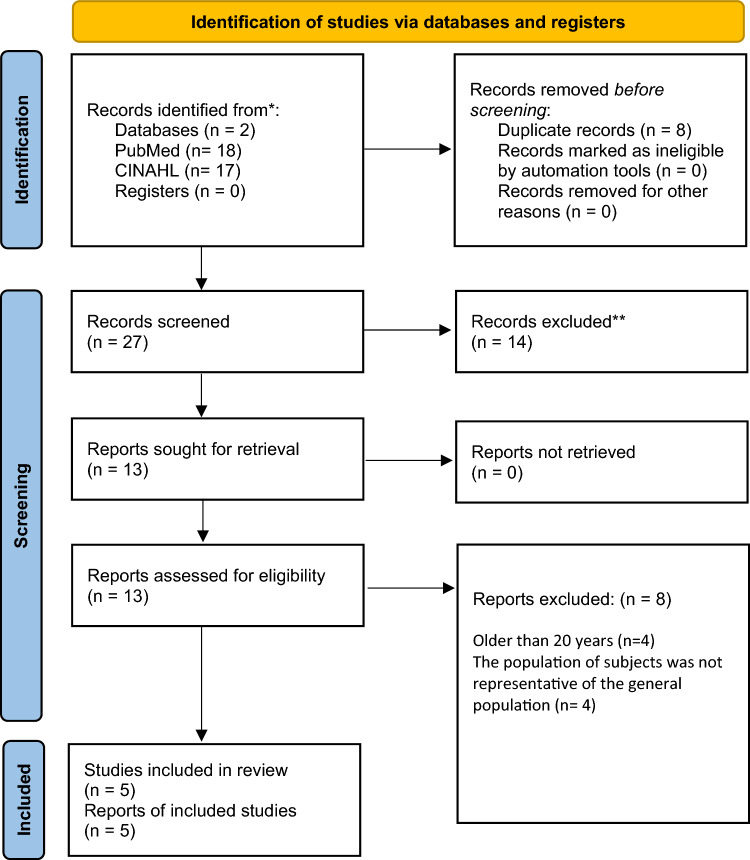
PRISMA Diagram

Literature searches were completed in PubMed and CINAHL databases using the following search terms: superior mesenteric artery syndrome (SMA), Wilkie’s syndrome, duodenal compression, scoliosis, idiopathic scoliosis, spinal fusion, spine surgery, body mass index, and underweight. Studies were excluded if subjects had a spinal deformity other than scoliosis, surgical intervention not for idiopathic scoliosis, or were in languages other than English. Studies older than twenty years were excluded. Figure 5 shows an overview of the search strategy and results. The database searches yielded thirty-five records. Duplicate records were removed, and twenty-seven records were screened by reviewing the titles and abstracts. Fourteen articles were excluded, and thirteen records were sought for retrieval. Eight articles were deemed ineligible for the following reasons: included SMA syndrome outcomes for surgery other than idiopathic scoliosis correction or study subjects were not representative of the general population (*n* = 4), or the study was older than 20 years (*n* = 4) The references from the five remaining original research articles were examined for other relevant articles that were not discovered during the database searches in PubMed and CINAHL An overview of the five remaining research articles reviewed is in Table 1.

**Table 1:** Summative table

Author, Year, Study Design,	Study Purpose	Study Population (Demographics)	Intervention and Settings	Outcome	Conclusions/ Results	Limitations of Findings
Shah, et.al. 2003 (19) Retrospective Chart Review	To investigate the relationship between weight and height and the likelihood of developing SMA syndrome following scoliosis surgery	Intervention: n = 6 patients developed SMA syndrome following scoliosis correction Scoliosis type: AIS n = 5 Friedrieich ataxia n = 1 Females n = 3 (mean age = 13 years) Males n = 3 (mean age 17 years) Time of SMA diagnosis Mean weight-for-height = 3rd percentile Control: n = 16 SMA syndrome-free patients who underwent scoliosis correction Mean weight-for-height = 49th percentile	1972–1995 Retrospective chart review of the presurgical weight-for-height percentile of patients that developed SMA syndrome after surgical correction of scoliosis against controls (N = 17)	n = 5 SMA syndrome diagnosis made 9–31 days (mean 17 days) post spinal fusion n = 1 SMA syndrome diagnosis was made 214 days post-spinal fusion Weight-for-height for the Intervention group vs control (age-matched) was significantly lower (P < 0.05) Two out of five had presurgical weight-for-height on the 25th percentile	Height is increased following spinal fusion Acute increase in height and postoperative weight loss increases the risk of developing SMA syndrome Weight-for-height ≤ 5th percentile can compromise the antimesenteric angle increasing the risk for vascular compression of the duodenum	Presurgical weight-for-height was only available for two out of six patients Scoliosis curve pattern was not obtained
Zhu and Qui, 2005(21) Prospective Chart Review	To investigate risk indicators, and common clinical presentation of SMA syndrome after scoliosis surgery	Developed SMA syndrome: n = 7 (0.01%) Scoliosis type: AIS n = 7 Curve pattern: Thoracic n = 5 Thoracolumbar n = 2 Kyphosis: Kyphosis n = 2 Hyperkyphosis n = 4 Females n = 4 (mean 14 years) Males n = 3 (mean age 15 years) Mean curve correction = 66 degrees	1997–2003 Prospective review (N = 640) of all scoliosis correction patients	SMA syndrome diagnosis was made 5 days post spinal fusion Mean weight-for-age = 14th percentile (range 5-25th percentile) Mean height-for-age = 23rd percentile (range 5-50th percentile)	Weight-for-age < 25th percentile and height-for-age < 50th percentile are risk factors for SMA syndrome	
Smith, et al. 2007(20) Retrospective Chart Review	To determine if BMI is a useful parameter to assess the risk of developing SMA syndrome following spinal infusion for scoliosis	Intervention: n = 5 patients Scoliosis type: AIS n = 3 NMS n = 1 Decompressed syrinx n = 1 Curve pattern: Thoracic n = 1 Thoracolumbar n = 2 Double major, thoracic, lumbar n = 2 Females n = 3 Males n = 2 Mean Pre-op BMI = 17.5 kg/m^2^ Mean Post-op BMI = 15.3 kg/m^2^ Mean curve correction = 75.4 degrees Control: n = 18 patients Mean Pre-op BMI = 21.7 kg/m^2^ Mean curve correction = 64.5 degrees	2000–2003 Retrospective chart review of pre- and post-op BMI of patients that developed SMA syndrome following spinal infusion for scoliosis correction compared to age-matched controls	Pre-op BMI of the control group was significantly higher than the intervention (P < 0.05) No difference was found between curve correction	BMI < 18 kg/m^2^ is a risk factor for developing SMA syndrome Immediate increase in height post-op and post-op decreased oral intake and weight loss may be the cause Clinicians should consider weight gain prior to surgery or immediate post-op enteral or parenteral nutrition	Post-op BMI was not available for the control group
Kim et al., 2008(18) Retrospective Chart Review	To determine the incidence and clarify risk factors associated with SMA syndrome after scoliosis surgery	Developed SMA n = 9 (7.6%) Scoliosis type: AIS n = 4 NMS n = 5 Mean age: SMA syndrome n = 15 years (± 3.6 years) Non SMA syndrome n = 15 years (± 4.4 years) Mean BMI: SMA syndrome = 14.6 kg/m^2^ (± 3.9 kg/m^2^) Non-SMA syndrome = 18.5 kg/m^2^ (± 3.5 kg/m^2^) Number of BMI-for-age < 5th Percentile: SMA syndrome group = 6 (66.7%) Non SMA syndrome group = 32 (29.4%) Mean increase in trunk height SMA syndrome = 4.5 cm (± 4.8 cm) Non SMA = 2.3 cm (± 2.1 cm) Mean curve correction SMA syndrome = 51 degrees Non SMA syndrome = 52 degrees	2001–2007 Retrospective chart review (N = 118) of all scoliosis correction patients	BMI for SMA syndrome group was significantly lower (P < 0.001) SMA syndrome group had a greater increase in trunk height, not statistically significant	Risk factors for SMA syndrome included: 1) Imbalance of height-to-weight exacerbated by an abrupt increase in trunk length and height 2) BMI-for-age < 5th percentile BMI-for-age percentile is more predictive of SMA syndrome risk than absolute BMI	Six out of 9 SMA syndrome patients were diagnosed by symptoms, not radiographic confirmation of obstruction Scoliosis curve pattern was not obtained
Louie et al. 2017(35) Case Report Review	Review of case reports to identify unique features related to the development of SMA syndrome after scoliosis correction	Nineteen case reports identified Scoliosis type: AIS n = 19 Curve pattern: Thoracic n = 5 Thoracolumbar n = 7 Double major, thoracic, thoracolumbar n = 3 Double major, thoracic, lumbar n = 4 Kyphosis n = 1	Case reports 1971–2017	Average postoperative weight loss was 6.2 pounds after the first week BMI-for-age < 25th percentile	Of the 19 case reports over the last 45 years, postoperative weight loss and BMI-for-age < 25th percentile are risk factors for SMA syndrome	

### Literature review

Shah et al. conducted a retrospective chart review to investigate the relationship between weight and height and the likelihood of developing SMA syndrome following scoliosis surgery. [23] The medical records of all patients who underwent any surgery and developed SMA syndrome at the Children’s Hospital of Pittsburgh between 1972 and 1995. [23] The patients identified (*N* = 17) were further divided into two groups, non-orthopedic (*n* = 11) or orthopedic, and underwent scoliosis surgery (*n* = 6). [23] Data collected included gender, age, scoliosis type, preoperative height and weight, preoperative gender-specific weight-for-height percentiles, postoperative height and weight, postoperative gender-specific weight-for-height percentiles, and the postoperative day the diagnosis of SMA syndrome was made. [23] Patient characteristics of individuals who developed SMA syndrome were compared against age-matched controls that did not develop SMA syndrome. [23] Of the six patients that underwent scoliosis surgery and developed SMA syndrome, five had adolescent idiopathic scoliosis and one had scoliosis secondary to Friedreich ataxia [*n* = 6, females *n* = 3 (mean age 13 years), males *n* = 3, (mean age 17 years)] with a mean presurgical weight-for-height on the 25th percentile and mean postoperative weight-for-height on the 3rd percentile. [23] The curve pattern in all six patients was not described [23]. The diagnosis of SMA syndrome was made between postoperative days 9–31 [*n* = 5, mean 17 days] and one patient had a late presentation with the diagnosis made on postoperative day 214. [23] Sixteen aged-match controls were included, with a mean presurgical weight-for-height on the 49th percentile. [23] The weight-for-height percentile for the SMA syndrome group was significantly lower (*P* < 0.05) than the weight-for-height percentile of the age-matched controls. [23] The authors noted the patients’ height increased as a result of the scoliosis surgery, which decreased the postoperative weight-for-height percentile as expected. [23] They also stated that postoperative weight loss is a complication that often occurs as part of the recovery process. [23] They concluded this acute increase in height and postoperative weight loss increases the risk of developing SMA syndrome. [23] Furthermore, a weight-for-height at or below the 5th percentile can compromise the aortomesenteric angle, increasing the risk for vascular compression of the duodenum [23].

Zhu and Qui conducted a prospective study to investigate risk indicators and common clinical presentation of SMA syndrome after scoliosis surgery. [25] The medical records [*N* = 640] of all patients with adolescent idiopathic scoliosis who underwent scoliosis surgery at Drum Tower Hospital, Nanjing University Medical School between 1997 and 2003 were prospectively evaluated to identify patients who developed SMA syndrome. [25] Data collected included gender, age, scoliosis type, curve pattern, gender-specific weight-for-age percentiles, gender-specific height-for-age percentiles, and the postoperative day the diagnosis of SMA syndrome was made. [25] Patient characteristics of individuals who developed SMA syndrome were compared against gender and age-matched controls who did not develop SMA syndrome. [25] Out of 640 patients, seven (0.1%) developed SMA syndrome [*n* = 7, females *n* = 4 (mean age 14 years), males *n* = 3, (mean age 15 years)] with a mean weight-for-age on the 14th percentile (5-25th percentile) and a mean height-for-age on the 23rd percentile (5-50th percentile). [25] Five out of the seven patients had a thoracic curve and the other two had a thoracolumbar curve. Additionally, six of the patients also had kyphosis; four had hyperkyphosis and two had kyphosis. [25] Diagnosis of SMA syndrome was, on average, made on postoperative day 5. [25] When compared against gender and age-matched controls for weight-for-age and height-for-age, the authors noted patients who developed SMA syndrome weighed less, but height-for-age was above the mean. [25] The authors also noted six of the seven patients who developed SMA syndrome had a thin habitus. [25] They concluded post-operative weight-for-age less than the 25th percentile, postoperative height-for-age less than the 50th percentile, and sagittal kyphosis are risk factors for the development of SMA syndrome [25].

Smith et al. conducted a retrospective chart review to determine if BMI was a practical parameter to assess the risk of developing SMA syndrome following spinal fusion for scoliosis. [24] The medical records of all patients who underwent scoliosis surgery at Connecticut Children’s Medical Center between 2000 and 2003 were reviewed to compare the preoperative and postoperative BMI of patients who developed SMA syndrome following scoliosis surgery compared to age-matched controls who did not develop SMA syndrome. [24] Five patients were identified, three had adolescent idiopathic scoliosis, one had neuromuscular scoliosis and one had a decompressed syrinx [curve pattern: thoracic curve (*n* = 1), thoracolumbar curve (*n* = 2), double major curve thoracic and lumbar (*n* = 2)]. [24] Data collected included age, scoliosis type, curve pattern, preoperative and postoperative weight and height (to calculate BMI), and postoperative day the diagnosis of SMA syndrome was made. [24] The five patients identified who developed SMA syndrome [*n* = 5, females *n* = 3 (mean age 15 years), males *n* = 2, (mean age 15 years)] had a mean preoperative BMI of 17.5 kg/m^2^ and mean postoperative BMI of 15.3 kg/m. [24] Eighteen age [*n* = 18] matched controls were identified with a mean preoperative BMI of 21.7 kg/m^2^. [24] The authors concluded a preoperative BMI of less than 18 kg/m^2^. should be considered a risk factor for the development of SMA syndrome. [24] Moreover, the authors concluded the immediate increase in postoperative height along with postoperative decreased oral intake and weight loss may be the cause. [24] They suggested clinicians should consider recommending weight gain before surgery or immediate post-op enteral or parenteral nutrition may be beneficial in underweight individuals [24].

Kim et al. conducted a retrospective chart review to determine the incidence and risk factors associated with SMA syndrome after scoliosis surgery. [22] The medical records [*N* = 118] of all patients who underwent scoliosis surgery at Young-Dong Severance and Bundang Cha Hospitals between 2001 and 2007 were reviewed to identify patients who developed SMA syndrome. [22] Eighty patients had adolescent idiopathic scoliosis, and 38 had neuromuscular scoliosis. [22] The patients were divided into two groups, SMA syndrome and non-SMA syndrome, and characteristics were compared. [22] Nine patients were identified, four with adolescent idiopathic scoliosis and five with neuromuscular scoliosis, curve pattern was not included [22], Data collected included gender, age, scoliosis type, height, and weight (to calculate BMI and BMI-for-age percentiles), postoperative height increase (cm), and postoperative day the diagnosis of SMA syndrome was made. [22] Among the nine patients that developed SMA syndrome [(*n* = 9 (7.6%), mean age = 15 years (± 3.6 years), mean BMI = 14.6 kg/m^2^ (± 3.9 kg/m^2^), BMI-for-age < 5th percentile = 6 (66%), mean increase in height = 4.5 cm (± 4.8 cm)]. [22] The remaining 109 patients, non-SMA syndrome, served as the control group [mean age = 15 years (± 4.4 years), mean BMI = 18.5 kg/m^2^ (± 3.5 kg/m^2^), percentile BMI-for-age < 5th percentile = 32 (29.4%), mean increase in height = 2.3 cm (± 2.1 cm). [22] The authors found the BMI for the SMA syndrome group was significantly lower than the non-SMA syndrome control group (P < 0.001). The SMA syndrome group had a greater postoperative increase in trunk height than the non-SMA syndrome control group but the difference between groups was not statistically significant. [22] The authors concluded the BMI-for-age percentile is more predictive of SMA syndrome risk than absolute BMI values. [22] They also concluded that risk factors for the development of SMA syndrome are a BMI for age less than the 5th percentile and abrupt postoperative height increases exacerbated by weight loss [22].

Louie et al. conducted a review of case reports from the last 45 years to identify unique features related to the development of SMA syndrome after scoliosis correction. [40] Nineteen case reports were identified between 1971 and 2017. [40] All the patients had adolescent idiopathic scoliosis [curve pattern: thoracic (*n* = 5), thoracolumbar (*n* = 7), double major curve, thoracic and thoracolumbar (*n* = 3), double major curve, thoracic and lumbar (*n* = 4), and kyphosis (*n* = 1) [40]. BMI-for-age percentile was found to be less than the 25th percentile and the average postoperative weight loss was 6.2 pounds after the first week. [40] The author concluded that having a BMI-for-age percentile less than the 25th percentile combined with postoperative weight loss should be considered risk factors for the development of SMA syndrome [40].

## Discussion

Four out of the five studies showed an association between being underweight or having a low BMI or BMI-for-age percentile, and the risk of developing SMA syndrome after scoliosis surgery. [22–25] The case report and literature review by Louie et al. show that despite changes in surgical technique, SMA syndrome after scoliosis surgery continues to be a nutritional consequence. [40] All five studies show that being underweight compounded by a surgical increase in height and postoperative weight loss puts patients at risk for the development of SMA syndrome. [22–25, 40] To date, there are no studies that focus on preoperative nutrition strategies for weight gain for the prevention of developing SMA syndrome following scoliosis surgery. Although limited, published nutrition intervention strategies all focus on the management of SMA syndrome after it has been diagnosed [3, 15, 41].

### Limitations

As stated by all authors, prospective and retrospective chart reviews have their limitations, including absent or inaccurate data and no method to confirm data. [22–25, 40] In all the prospective and retrospective chart reviews, there was incomplete or missing data. [22–25] Another limitation is that SMA syndrome can be challenging to diagnose, and mild symptoms may get overlooked. [22, 40] Additionally, SMA syndrome diagnosis is often made without radiographic confirmation. [3, 6, 9] This was pointed out in the study by Kim et al., in which six out of the nine patients were diagnosed with SMA syndrome based on symptoms, not radiographic confirmation. [22] Due to the subjective nature of the symptoms, SMA syndrome may be underreported in the literature.

### Implications for practice

To date, published nutritional strategies have focused on the management of SMA syndrome once the diagnosis has been made, as opposed to developing nutrition strategies to prevent SMA syndrome from developing. [3, 15] For example, Albano et al. presented a case of a nineteen-year-old underweight female who presented with an acute duodenal obstruction and developed SMA syndrome. [41] Nasogastric decompression was rapidly initiated followed by nasogastric enteral nutrition resulting in an increase in body weight and resolution of SMA syndrome. [41] SMA syndrome often results soon after surgery, and Albano et al. were able to show that immediately increasing body weight with enteral nutrition can quickly resolve SMA syndrome and potentially prevent surgery. [41] Based on the findings in this literature review, more attention should be placed on identifying underweight patients and increasing their preoperative body weight to prevent a reduction in both the mesenteric fat pad and aortomesenteric angle, thus decreasing the likelihood of SMA syndrome.

It is important to note that while underweight individuals are at an increased risk, SMA syndrome can occur in individuals of any body type and is not exclusive to those who are underweight. [1, 3, 7, 41] Nonetheless, underweight individuals have a higher susceptibility to the development of SMA syndrome. [22–26, 40] Close monitoring, early detection, and tailored preoperative and postoperative nutrition strategies are essential to minimize the risk and effectively manage SMA syndrome in these patients.

Surgical complications have a substantial impact on healthcare costs [42, 43]. Complications increase the length of stay and readmissions are now being used as indicators of quality care and can negatively impact hospital reimbursement [42–44]. Tarant et al., evaluated the association between having a low BMI and pre and post-surgical factors following spinal fusion for adolescent idiopathic scoliosis [35]. They found the development of postoperative ileus was independently associated with a low BMI [35]. Postoperative ileus, a primary feature of SMA syndrome, leads to significant morbidity and is associated with increased hospital costs and length of stay [35, 43, 45]. Boylan et al., in a retrospective comparative study, found an increased length of stay following adolescent idiopathic scoliosis surgery was associated with an increased cost, increased risk of all-cause readmission and return to the operating room [43]. An additional one day in the hospital was associated with $11, 033 in insurance charges, $5198 in hospital costs, a 28% increased risk of all-cause 90-day readmission, and a 57% increased risk of returning to the operating room within 90 days [43]. They concluded there is a cost–benefit to having protocols that decrease complication risk and length of stay [43].

Although rare, SMA syndrome is a known complication of scoliosis surgery [9, 25]. As shown, being underweight increases the risk of developing SMA syndrome [22–25, 40]. In addition to SMA syndrome pre-surgical underweight (malnutrition) is associated with other post-operative complications including increased length of stay, delayed or poor wound healing, and increased risk of infection [35, 42, 43, 45–47]. Therefore, strategies should be put in place to minimize all complications associated with low body weight and or malnutrition [44, 47–49].

More recently, the development of Enhanced Recovery After Surgery (ERAS) supports the practice of pre-surgical nutrition screening to identify malnourished patients to improve nutrition status prior to surgery to reduce associated postoperative complications [45, 46]. This strategy is well established in the adult spinal surgery population and continues to emerge in pediatric care [43, 45–47]. The current recommendation is to provide pre-surgical nutrition interventions to those identified as malnourished. Xu et al. in an RCT of spinal surgery patients, reported nutritional interventions during the preoperative and perioperative period in undernourished patients reduced length of stay, decreased incidence of electrolyte disturbances, and resulted in higher postoperative albumin levels compared to controls [50]. Scott et al. pointed out the potential gain for patients, by implementing a nutrition screen process and timely pre-operative nutrition interventions that are continued through the operative and post-operative period is simple, safe, and most cost-effective to improve surgical outcomes [44].

As evident by the lack of literature, there is limited clinical guidance for the pre-surgical identification and nutritional management of underweight or malnourished pediatric patients undergoing scoliosis surgery [47]. Adequate pre-surgical nutrition screening and interventions are vital yet underrecognized aspects of improving optimal surgical outcomes in these pediatric patients [43, 47–49].

A rapid increase in height compounded by post-operative weight loss can also put patients at risk for developing SMA syndrome. Screening by BMI alone may not be the most comprehensive method for identifying “at-risk “patients. Preoperative radiographic screening of all patients using ultrasound may be a low radiation, viable option to measure the aortomesenteric angle since an aortomesenteric angle near or less than 25 degrees can be indicative of a reduced mesenteric fat pad. [4–6] Therefore, any patient found to have a preoperative aortomesenteric angle at or less than 25 degrees could be counseled on strategies for gaining weight to increase the mesenteric fat pad, thus increasing the aortomesenteric angle and reducing SMA syndrome risk.

**Fig. 6:**
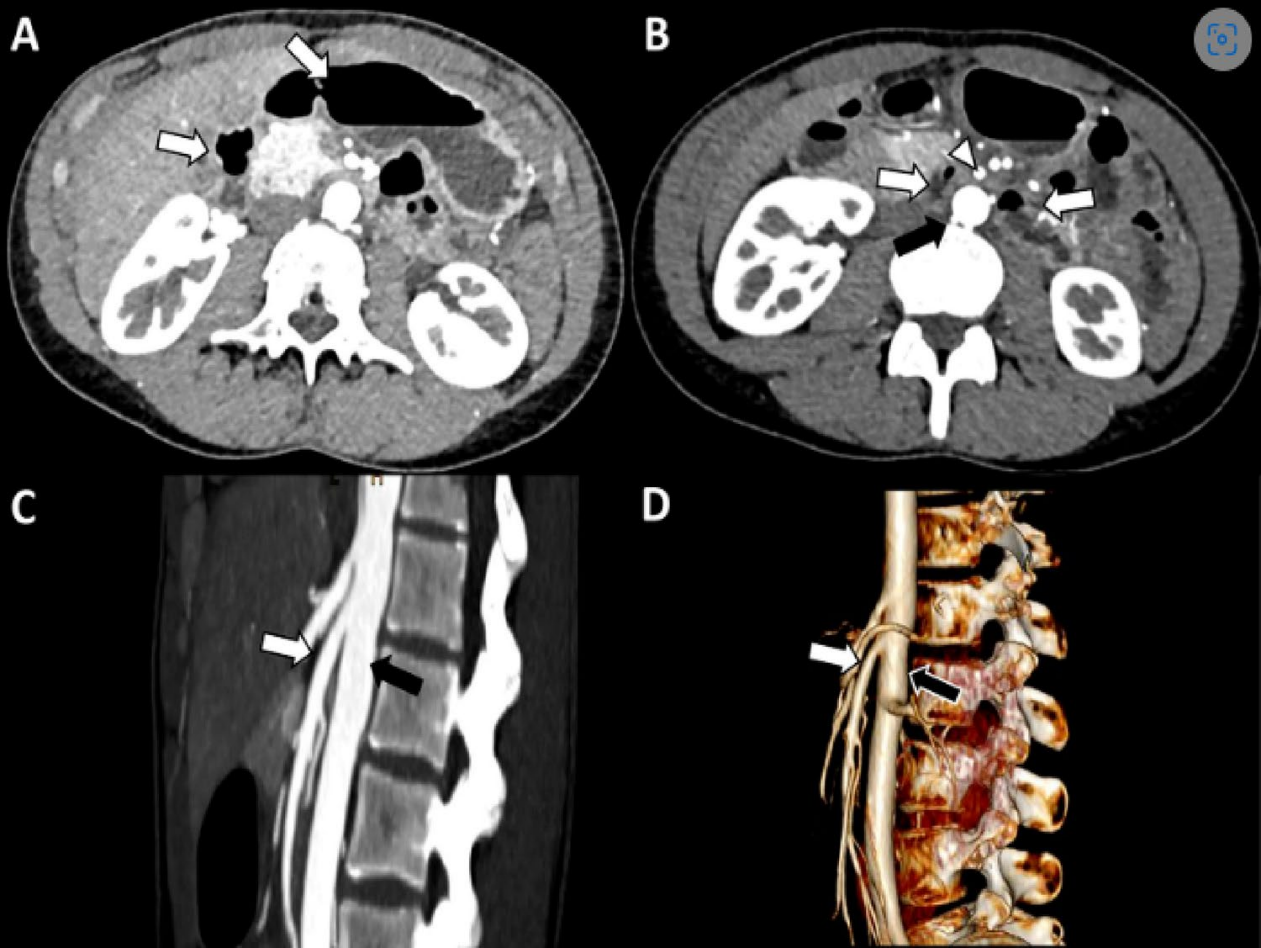
Superior mesenteric artery syndrome. Axial contrast-enhanced CT images at the level of the upper abdomen. **A** Mild distension of the stomach and proximal duodenum (white arrows). **B** Compression of the third portion of the duodenum (white arrows) between the superior mesenteric artery (white arrowhead) and abdominal aorta (black arrow). **C** Sagittal images show reduced angle and distance between the superior mesenteric artery (white arrow) and abdominal aorta (black arrow) (aortomesenteric angle of 18°, aortomesenteric distance of 5 mm). **D** Sagittal 3D reconstruction again shows the reduced angle between the superior mesenteric artery (white arrow) and abdominal aorta (black arrow)

**Fig. 7:**
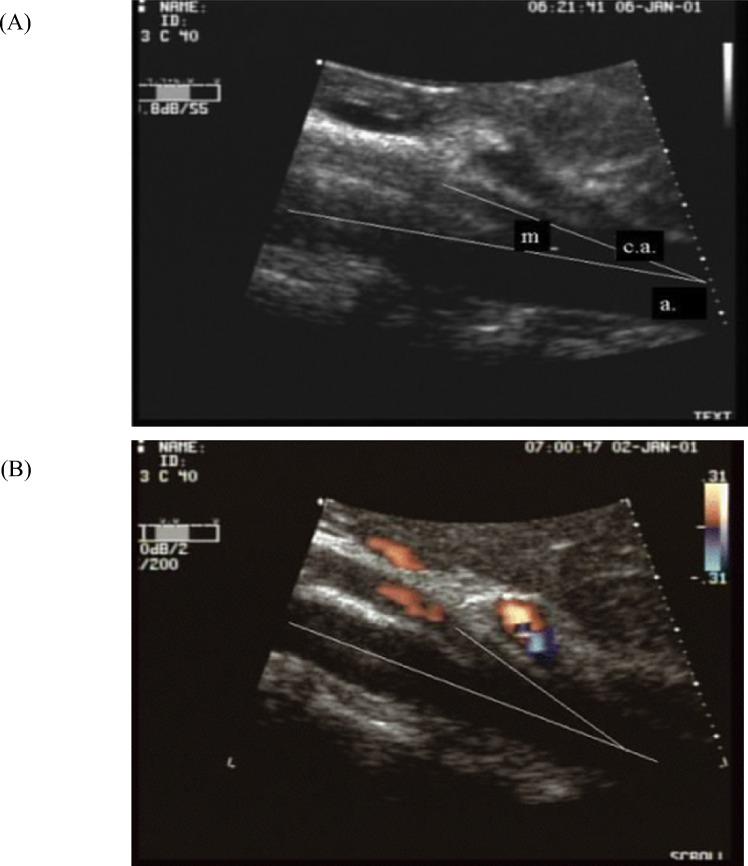
Superior mesenteric artery syndrome. **A** Ultrasound of supine patient; measuring aortomesenteric artery at 14 degrees. **B** Echo Color Doppler of patient lying down: measuring aortomesenteric artery at 21 degrees

Currently, a CT scan is the gold standard for measuring the aortomesenteric angle, but frequent CT scans would put the patient at risk for frequent radiation exposure (Fig. 6). [3, 11] Ultrasound imaging may be a screening option that eliminates radiation exposure. [12, 13] In a study by Neri et al., 950 patients underwent an abdominal ultrasound followed by an Echo Color Doppler of the epigastric area (Fig. 7). [12] They were able to diagnose SMA syndrome in 29 patients who then underwent confirmation via CT scan. [12] Another study by Mauceri et al. performed abdominal ultrasounds followed by an Echo Color Doppler on 460 patients, identifying 20 patients with aortomesenteric angles of less than 25 degrees with 18 of them having a duodenal compression. [13] Both studies concluded ultrasound is a rapid, repeatable, noninvasive, low-cost diagnostic method for measuring the aortomesenteric angle and can be a useful epidemiological screen to determine the presence of a reduced angle or SMA syndrome. [12, 13] Although limited research has been conducted to date, this warrants further exploration.

Finally, there is limited data regarding scoliosis type and curve pattern. The type of scoliosis and risk of developing SMA syndrome is not well described in the literature. However, regarding curve pattern, Braun et al. and Zhu et al. both identified having a stiffer thoracic curve (< 60% correction on bending radiographs), a laterally displaced lumbar curve (Lenke B or C), and kyphosis as risk factors for developing SMA syndrome [25, 30]. These factors also warrant further exploration.

## Conclusion

SMA syndrome is a rare complication following scoliosis surgery and the incidence is low. [21] However, if symptoms are not recognized, the result can cause lasting negative effects on health outcomes including gastric pneumatosis, duodenal obstruction, gastric perforation, or death. [10, 21] The ability to recognize risk factors and patient characteristics is key to the prevention of SMA syndrome. [10, 22–24, 40] Based on the literature review, being underweight or having a low BMI or BMI-for-age percentile puts patients at risk for developing SMA syndrome following corrective surgery for scoliosis. [1, 2, 23, 40]

### Implications for research

Future research should focus on not only identifying underweight patients preoperatively but also developing nutrition strategies that further reduce the likelihood of developing SMA syndrome. Prospective studies are needed that focus on preoperative nutrition counseling for weight gain in the prevention of SMA syndrome. To date, there are no published studies that focus on nutrition strategies for the prevention of SMA syndrome following scoliosis surgery.

Future collaborative research efforts between orthopedists, registered dietitians, gastroenterologists, and radiologists will be instrumental in advancing the knowledge of SMA syndrome in underweight individuals and developing more effective preventive treatment strategies.

## Data Availability

The data that support the findings of this study are available from the corresponding author, upon reasonable request.
